# Inequalities in the health survey using validation question to filter insufficient effort responding: reducing overestimated effects or creating selection bias?

**DOI:** 10.1186/s12939-019-1030-2

**Published:** 2019-08-22

**Authors:** Jingjing Lu, Feng Wang, Xiaomin Wang, Leesa Lin, Weiyi Wang, Lu Li, Xudong Zhou

**Affiliations:** 1 The Institute of Social and Family Medicine, School of Medicine, Zhejiang University, 866 Yuhangtang Rd, Hangzhou, Zhejiang 310058 People’s Republic of China; 2 Department of Public Health, Environments and Society, Faculty of Public Health and Policy, London School of Hygiene & Tropical Medicine, 15-17 Tavistock Place, London, WC1H 9SH UK; 3 Department of Global Health and Social Medicine, Harvard Medical School, 641 Huntington Avenue, Boston, MA 02215 USA

**Keywords:** Insufficient effort responding, Validation question, Selection bias

## Abstract

**Background:**

The presence of insufficient effort responding participants (IERPs) in a survey can produce systematic bias. Validation questions are commonly used to exclude IERPs. Participants were defined as IERPs if responding inconsistently to two matched validation questions, and non-insufficient effort responding participants (non-IERPs) if responding consistently. However, it has not been tested whether validation questions themselves could result in selection bias.

**Methods:**

This study was a cross-sectional survey conducted in Guangxi, China. Participants’ intentions to use antibiotics for their children when they have self-limiting diseases, including sore throat, cold, diarrhea, and fever, were measured. The Chi-square tests were used to compare the socio-economic status (SES) between non-IERPs and IERPs. Logistic regression was adopted to test the association between intentions to misuse antibiotics and groups (non-IERPs, IERPs with high SES, and IERPs with low SES).

**Results:**

Data with 3264 non-IERPs and 1543 IERPs were collected. The results showed IERPs had a lower education level (χ2 = 6.100, *p* = 0.047) and a higher proportion of rural residence (χ2 = 4.750, *p* = 0.030) compared with non-IERPs. Rural IERPs reported significantly higher rates of intentions to misuse antibiotics when their children have a sore throat (OR = 1.32; 95% CI = 1.11,1.56; *p* < 0.01), cold (OR = 1.33; 95%CI = 1.13,1.58; *p* < 0.01), diarrhea (OR = 1.46; 95%CI = 1.20,1.77; *p* < 0.001), and fever (OR = 1.22; 95% CI = 1.04,1.43; *p* < 0.05) compared with non-IERPs. IERPs living in urban areas reported significantly lower rates of intentions to use antibiotics when their children have a sore throat (OR = 0.76; 95%CI = 0.62,0.93; *p* < 0.01) compared with non-IERPs. IERPs with lower levels of education reported significantly higher rates of intentions to use antibiotics when their children have a sore throat (OR = 1.19; 95%CI = 1.02,1.39; *p* < 0.05), cold (OR = 1.43; 95% CI = 1.23,1.66; *p* < 0.001), diarrhea (OR = 1.38; 95%CI = 1.15,1.64; *p* < 0.01), and fever (OR = 1.25; 95% CI = 1.09,1.44; *p* < 0.01) compared with non-IERPs. IERPs with higher education levels reported significantly lower rates of intentions to use antibiotics when their children have a sore throat (OR = 0.72; 95% CI = 0.56,0.94; *p* < 0.05), cold (OR = 0.66; 95% CI = 0.51,0.86; *p* < 0.01), and fever (OR = 0.74; 95% CI = 0.60,0.92; *p* < 0.01) compared with non-IERPs. IERPs with low-income reported significantly higher rates of intentions to use antibiotics when their children have a cold (OR = 1.36; 95% CI = 1.13,1.64; *p* < 0.01) and diarrhea (OR = 1.30; 95% CI = 1.05,1.62; *p* < 0.05) compared with non-IERPs.

**Conclusions:**

Using validation questions to exclude IERPs can result in selection bias in which participants with lower socio-economic standing and poor antibiotic use intentions were disproportionately excluded.

## Background

Research in social sciences often relies upon the motivation of participants to provide authentic data. Unmotivated participants may undermine the detection of real differences through response sets such as insufficient effort responding, or invalid responding. Failure to screen for, and exclude, potentially insufficient effort responses increases noise in data [1, 2]. Insufficient effort responding negates the usefulness of responses and introduces substantial error variance to analyses. Scholars suggest insufficient effort responses may be motivated by a lack of preparation, reactivity to observation, lack of motivation to cooperate, disinterest, or fatigue [3, 4]. Contrary to the common assumption that insufficient effort responses seldom happen and are unlikely to threaten data integrity, their prevalence has been reported to be as high as 40%, [5] and rates of merely 5% have been shown to exaggerate or mute associations found between variables [6].

Validation questions are widely used in both clinical and research contexts to screen for insufficient effort responses.[7] One simple strategy for validity screening is employing screening questions, for example, asking participants whether they are telling the truth on the survey or whether they are reading this survey very carefully. Furthermore, the inconsistency approach is also documented as identifying insufficient effort responses [8, 9]. This approach typically uses matched item pairs and compares the response on one item to the response on the other [10]. Item pairs are created in three ways, including 1) direct item repetition, 2) rational selection, and 3) empirical selection. Scholars also recommend that survey researchers design very similar questions in different places on a questionnaire to check against insufficient effort responding participants (IERPs).[11] Participants were defined as IERPs if responding inconsistently to these two matched questions, and non-insufficient effort responding participants (non-IERPs) if responding consistently.

IERPs aren’t always the result of inattentiveness, but also might be due to limited health literacy and numeracy skills in the context of understanding health information [12]. This may be especially true for people living in rural areas where access to health messages is limited and education levels are low [13]. Therefore, researchers may be disproportionally excluding the socio-economically disadvantaged in their attempts to limit IERPs. However, no study to our knowledge has performed such an exploration.

Before beginning this study, we conducted a survey on antibiotic rational use knowledge and behaviors among young Chinese parents in one eastern and one western provinces with vast disparities in social and economic development. Although we used the same questionnaire and validation question, we found a huge gap in the proportion of non-IERPs and IERPs between the eastern developed province and the western developing province. Data from this study came from a cross-sectional study conducted in Guangxi Zhuang autonomous region and Zhejiang province. For this investigation, we used data from Guangxi because an unusual amount of IERPs emerged during data collection process. We aimed to investigate whether the presence of a validation question reduced the overestimated effects or created selection bias in developing areas with relatively low social and economic development.

## Methods

This study was a cross-sectional survey of antibiotic use related knowledge and behaviors among parents with children aged 0–14 years in Guangxi Zhuang autonomous region, a southwestern province of China. Guangxi’s 2017 nominal gross domestic product per capita was about 38,102 RMB (US$6047.9) and ranked 27th among the 31 provinces in China (excluding Macao Special Administrative Region, Hong Kong Special Administrative Region, and Taiwan Province) [14].

### Data collection

The survey was conducted from November to December 2017. Twelve sites (six in urban areas and six in rural areas) were selected using the stratified random cluster sampling method: three community health service centers’ vaccination sites targeting parents with children under 3 years old, six kindergartens targeting parents with children aged 4–7 years, and three primary schools targeting parents with children aged 8–14 years. Considering the differences in school size and population density between urban and rural areas in Guangxi, two urban community health service centers’ vaccination sites, two urban kindergartens, one urban primary school, one rural community health service centers’ vaccination site, four rural kindergartens, and two rural primary schools were randomly selected from the roster of the city Health Bureau. The vaccination site was a department specially set up for providing vaccinations in each community health service center. Chinese parents are required to have their children vaccinated in these community health service centers before enrolling them in kindergarten. We used the electronic questionnaire tool, Wen Juan Xing (Chinese Survey Monkey), to conduct the survey. Permission to conduct the survey was initially obtained from each vaccination site as well as kindergarten and primary school authorities.

The survey at kindergartens and primary schools was conducted with the assistance of school staff. Survey information packs (an envelope with a letter for parents, a simplified instruction guide and a printed QR code of the electronic questionnaire inside) were distributed to parents by school staff.

At the vaccination site, four investigators approached parents who had gotten their children (under 3 years old) vaccinated, explained the aim of our survey, disseminated the printed QR (quick response) code of the electronic questionnaire, and showed them how to complete the electronic questionnaire.

### Study variables

This questionnaire was developed based on one former antibiotic knowledge assessment on university students in China [15] and modified by qualitative interviews with stakeholders and experts in child antibiotic use. This questionnaire was finalized after a pilot test with 315 respondents to evaluate potential sources of response error and to validate the questionnaire.

Two sections of the questionnaire were analyzed in the present study: 1) socio-economic information, and 2) parents’ intentions to use antibiotics when their children have certain self-limiting diseases. Socio-economic status (SES) information included: residence, education level and household income per month. Education level referred to the highest education level of either parent. High school education or below was identified as “low-education” while college education or above was classified as “high-education.” Household income per month below 5000 RMB was identified as “low-income” and above 5000 RMB as “high-income” in the present study. The intentions to use antibiotics were assessed by asking parents if they intend to use antibiotics when their children have a sore throat/cold/diarrhea/fever. The following statement was identified as a “yes” answer for intentions to use antibiotics: “I’d like to apply antibiotics when my children were having a sore throat/cold/diarrhea/fever.” The following statements were identified as a “no” answer for intentions to use antibiotics: “I wouldn’t apply antibiotics when my children were having a sore throat/cold/diarrhea/fever.” and “I don’t know if I should apply antibiotics when my children were having a sore throat/cold/diarrhea/fever but I would like to take doctors’ advice.” The Cronbach’s Alpha was 0.822.

The validity status was assessed with a pair of matched statements embedded in the questionnaire: “Antibiotics are effective for viral infections.” and “Antibiotics are effective for children’s viral infections.” Participants could choose from “Yes”, “No”, and “I don’t know”. Participants were defined as IERPs if responding inconsistently to these two statements, and non-IERPs if responding consistently. The IERPs were then divided into high socio-economic groups and low socio-economic groups according to education level, residence, and household income.

### Study sample

The sample size was designed as 350 urban non-IERPs with children under 3 years old, 350 rural non-IERPs with children under 3 years old, 400 urban non-IERPs with children aged 4–7 years, 400 rural non-IERPs with children aged 4–7 years, 750 urban non-IERPs with children aged 8–14 years, and 750 rural non-IERPs with children aged 8–14 years.

A total of 4550 parents in the selected kindergartens and primary schools were invited to participate, of which 3679 (80.86%) completed the questionnaire, and 2556 (69.48%) were non-IERPs. A total of 1230 parents in the selected vaccination site were invited to participate during the survey period, of which 1128 (91.71%) completed the questionnaire, and 708 (62.41%) were non-IERPs. Thus, 4807 parents were included for the present study.

### Statistical analysis

Chi-square tests were conducted to compare the socio-economic status between IERPs and non-IERPs. Logistic regression models were adopted to examine the differences in intentions to misuse antibiotics between non-IERPs and IERPs with high and low SES. All analyses were performed using SPSS 20.0 version and assumed a statistical significance level of *p* < 0.05.

## Results

Across the 12 sites, out of 5780 eligible parents, 4807 (83.17%) completed the survey, of whom 1543 (32.10%) were IERPs and 3264 (67.90%) were non-IERPs. Descriptive statistics for the measurements are given in Table 1. Compared with IERPs, non-IERPs reported lower rates of intentions to use antibiotics when their children have a sore throat (χ2 = 23.92, *p* < 0.001), cold (χ2 = 21.75, *p* < 0.001), diarrhea (χ2 = 12.41, *p* = 0.002), and fever (χ2 = 11.10, *p* = 0.004). Participants identified as IERPs generally had a lower education level (χ2 = 6.100, *p* = 0.047) and a higher proportion of rural residence (χ2 = 4.750, *p* = 0.030), but showed no statistical difference in household income (χ2 = 5.160, *p* = 0.271).

**Table 1:** Descriptive statistics for participants stratified by non-IERP/IERP Groups

	Total sample *N* = 4807	Non-IERP group *N* = 3264	IERP group *N* = 1543	χ2	*P*
	N (%)	N (%)	N (%)		
Residence				4.750	0.030
Rural	2479 (51.57)	1648 (50.49)	831 (53.86)		
Urban	2328 (48.43)	1616 (49.51)	712 (46.14)		
Education level				6.100	0.047
Middle school or below	1789 (37.22)	1185 (36.31)	604 (39.14)		
High school	1481 (30.81)	1000 (30.64)	481 (31.17)		
College or above	1537 (31.97)	1079 (33.05)	458 (29.69)		
Household income per month				5.160	0.271
< 3000 RMB	1872 (38.94)	1238 (37.93)	634 (41.09)		
3001–5000 RMB	1577 (32.81)	1079 (33.06)	498 (32.27)		
5001–10,000 RMB	967 (20.12)	673 (20.62)	294 (19.05)		
10,001–20,000 RMB	282 (5.87)	196 (6.00)	86 (5.57)		
> 20,000 RMB	109 (2.27)	78 (2.39)	31 (2.01)		
Intentions to use antibiotics					
Sore throat				23.92	< 0.001
No	2770 (57.62)	1835 (56.22)	935 (60.60)		
Don’t know	831 (17.29)	624 (19.12)	207 (13.42)		
Yes	1206 (25.09)	805 (24.66)	401 (25.99)		
Cold				21.75	< 0.001
No	2805 (58.35)	1891 (57.94)	914 (59.24)		
Don’t know	759 (15.79)	567 (17.37)	192 (12.44)		
Yes	1243 (25.86)	806 (24.69)	437 (28.32)		
Diarrhea				12.41	0.002
No	3026 (62.95)	2040 (62.50)	986 (63.90)		
Don’t know	1014 (21.09)	730 (22.37)	284 (18.41)		
Yes	767 (15.96)	494 (15.13)	273 (17.69)		
Fever				11.10	0.004
No	2041 (42.46)	1364 (41.79)	677 (43.88)		
Don’t know	894 (15.60)	649 (19.88)	245 (15.88)		
Yes	1872 (38.94)	1251 (38.33)	621 (40.25)		

Table 2 shows the binary logistic regression analyses of participants’ intentions to use antibiotics. After controlling for residence, education level, and household income per month, IERPs reported significantly higher rates of intentions to use antibiotics when their children have a cold (OR = 1.17; 95% CI = 1.02,1.35; *p* < 0.05) and diarrhea (OR = 1.19; 95% CI = 1.01,1.40; *p* < 0.05).

**Table 2:** Binary logistic regression coefficients for participants’ intentions to use antibiotics on response status with adjustment for socio-economic status

	Sore throat OR(95%CI)	Cold OR(95%CI)	Diarrhea OR(95%CI)	Fever OR(95%CI)
Group				
Non-IERPs	1.00	1.00	1.00	1.00
IERPs	1.05 (0.91,1.20)	1.17 (1.02,1.35)*	1.19 (1.01,1.40)*	1.07 (0.95,1.21)
Residence				
Rural	1.00	1.00	1.00	1.00
Urban	0.68 (0.59,0.79)***	0.79 (0.68,0.92)***	0.71 (0.59,0.84)***	0.92 (0.81,1.05)
Education level				
Low-education	1.00	1.00	1.00	1.00
High-education	0.71 (0.60,0.84)***	0.46 (0.39,0.55)***	0.78 (0.65,0.95)*	0.72 (0.63,0.83)***
Household income per month				
Low-income	1.00	1.00	1.00	1.00
High-income	0.84 (0.70,0.99)*	0.87 (0.74,1.04)	1.03 (0.85,1.26)	1.01 (0.87,1.16)

*p < 0.05, **p < 0.01, ***p < 0.001

Table 3 shows the binary logistic regression analyses of participants’ intentions to use antibiotics when their children have a sore throat. After controlling for education level and household income, urban IERPs reported significantly lower rates of intentions to use antibiotics (OR = 0.76; 95%CI = 0.62,0.93; *p* < 0.01) while rural IERPs reported significantly higher rates (OR = 1.32; 95%CI = 1.11,1.56; *p* < 0.01) compared with non-IERPs. After controlling for residence and household income per month, IERPs with high-education reported significantly lower rates of intentions to use antibiotics (OR = 0.72; 95%CI = 0.56,0.94; *p* < 0.05) while IERPs with low-education reported significantly higher rates (OR = 1.19; 95%CI = 1.02,1.39; *p* < 0.05) compared with non-IERPs.

**Table 3:** Results of binary logistic regression models by participants’ intentions to use antibiotics in sore throat

	Model 1 OR(95%CI)	Model 2 OR(95%CI)	Model 3 OR(95%CI)
Group			
Non-IERPs	1.00	/	/
IERPs * Urban	0.76 (0.62,0.93)**	/	/
IERPs * Rural	1.32 (1.11,1.56)**	/	/
Group			
Non-IERPs	/	1.00	/
IERPs * High-education	/	0.72 (0.56,0.94)*	/
IERPs * Low-education	/	1.19 (1.02,1.39)*	/
Group			
Non-IERPs	/	/	1.00
IERPs * High-income	/	/	0.99 (0.83,1.19)
IERPs * Low-income	/	/	1.12 (0.92,1.35)
Residence			
Rural	/	1.00	1.00
Urban	/	0.65 (0.56,0.75)***	0.66 (0.57,0.76)***
Education level			
Low-education	1.00	/	1.00
High-education	0.66 (0.56,0.78)***	/	0.68 (0.58,0.80)***
Household income per month			
Low-income	1.00	1.00	/
High-income	0.78 (0.66,0.92)**	0.79 (0.67,0.94)**	/

*p < 0.05, **p < 0.01, ***p < 0.001

Table 4 shows the binary logistic regression analyses of participants’ intentions to use antibiotics when their children have a cold. After controlling for education level and household income, rural IERPs reported significantly higher rates of intentions to use antibiotics (OR = 1.33; 95%CI = 1.13,1.58; *p* < 0.01) compared with non-IERPs. After controlling for residence and household income per month, IERPs with high-education reported significantly lower rates of intentions to use antibiotics (OR = 0.66; 95%CI = 0.51,0.86; *p* < 0.01) while IERPs with low-education reported significantly higher rates (OR = 1.43; 95%CI = 1.23,1.66; *p* < 0.001) compared with non-IERPs. After controlling for residence and education level, IERPs with low-income reported significantly higher rates of intentions to use antibiotics (OR = 1.36; 95%CI = 1.13,1.64; *p* < 0.01) compared with non-IERPs.

**Table 4:** Results of binary logistic regression models by participants’ intentions to use antibiotics in cold

	Model 1 OR(95%CI)	Model 2 OR(95%CI)	Model 3 OR(95%CI)
Group			
Non-IERPs	1.00	/	/
IERPs * Urban	0.99 (0.81,1.20)	/	/
IERPs * Rural	1.33 (1.13,1.58)**	/	/
Group			
Non-IERPs	/	1.00	/
IERPs * High-education	/	0.66 (0.51,0.86)**	/
IERPs * Low-education	/	1.43 (1.23,1.66)***	/
Group			
Non-IERPs	/	/	1.00
IERPs * High-income	/	/	1.03 (0.87,1.23)
IERPs * Low-income	/	/	1.36 (1.13,1.64)**
Residence			
Rural	/	1.00	1.00
Urban	/	0.70 (0.61,0.81)***	0.79 (0.68,0.91)**
Education level			
Low-education	1.00	/	1.00
High-education	0.44 (0.38,0.53)***	/	0.46 (0.39,0.54)***
Household income per month			
Low-income	1.00	1.00	/
High-income	0.84 (0.71,0.99)*	0.77 (0.65,0.90)**	/

*p < 0.05, **p < 0.01, ***p < 0.001

Table 5 shows the binary logistic regression analyses of participants’ intentions to use antibiotics when their children have diarrhea. After controlling for education level and household income per month, rural IERPs reported significantly higher rates of intentions to use antibiotics (OR = 1.46; 95%CI = 1.20,1.77; *p* < 0.001) compared with non-IERPs. After controlling for residence and household income per month, IERPs with low-education reported significantly higher rates of intentions to use antibiotics (OR = 1.38; 95% CI = 1.15,1.64; *p* < 0.01) compared with non-IERPs. After controlling for residence and education level, IERPs with low-income reported significantly higher rates of intentions to use antibiotics (OR = 1.30; 95% CI = 1.05,1.62; *p* < 0.05) compared with non-IERPs.

**Table 5:** Results of binary logistic regression models by participants’ intentions to use antibiotics in diarrhea

	Model 1 OR(95%CI)	Model 2 OR(95%CI)	Model 3 OR(95%CI)
Group			
Non-IERPs	1.00	/	/
IERPs * Urban	0.89 (0.70,1.13)	/	/
IERPs * Rural	1.46 (1.20,1.77)***	/	/
Group			
Non-IERPs	/	1.00	/
IERPs * High-education	/	0.76 (0.56,1.04)	/
IERPs * Low-education	/	1.38 (1.15,1.64)**	/
Group			
Non-IERPs	/	/	1.00
IERPs * High-income	/	/	1.10 (0.89,1.35)
IERPs * Low-income	/	/	1.30 (1.05,1.62)*
Residence			
Rural	/	1.00	1.00
Urban	/	069 (0.58,0.82)***	0.73 (0.61,0.86)***
Education level			
Low-education	1.00	/	1.00
High-education	0.74 (0.61,0.89)**	/	0.80 (0.66,0.96)*
Household income per month			
Low-income	1.00	1.00	/
High-income	0.97 (0.80,1.18)	1.02 (0.84,1.23)	/

*p < 0.05, **p < 0.01, ***p < 0.001

Table 6 shows the binary logistic regression analyses of participants’ intentions to use antibiotics when their children have a fever. After controlling for education level and household income per month, rural IERPs reported significantly higher rates of intentions to use antibiotics (OR = 1.22; 95% CI = 1.04,1.43; *p* < 0.05) compared with non-IERPs. After controlling for residence and household income per month, IERPs with high-education reported significantly lower rates of intentions to use antibiotics (OR = 0.74; 95%CI = 0.60,0.92; *p* < 0.01) while IERPs with low-education reported significantly higher rates (OR = 1.25; 95%CI = 1.09,1.44; *p* < 0.01) compared with non-IERPs.

**Table 6:** Results of binary logistic regression models by participants’ intentions to use antibiotics in fever

	Model 1 OR(95%CI)	Model 2 OR(95%CI)	Model 3 OR(95%CI)
Group			
Non-IERPs	1.00	/	/
IERPs * Urban	0.92 (0.77,1.09)	/	/
IERPs * Rural	1.22 (1.04,1.43)*	/	/
Group			
Non-IERPs	/	1.00	/
IERPs * High-education	/	0.74 (0.60,0.92)**	/
IERPs * Low-education	/	1.25 (1.09,1.44)**	/
Group			
Non-IERPs	/	/	1.00
IERPs * High-income	/	/	1.02 (0.87,1.19)
IERPs * Low-income	/	/	1.15 (0.96,1.40)
Residence			
Rural	/	1.00	1.00
Urban	/	0.88 (0.78,1.01)	0.94 (0.82,1.06)
Education level			
Low-education	1.00	/	1.00
High-education	0.72 (0.63,0.83)***	/	0.73 (0.63,0.83)***
Household income per month			
Low-income	1.00	1.00	/
High-income	1.01 (0.88,1.16)	0.96 (0.83,1.11)	/

*p < 0.05, **p < 0.01, ***p < 0.001

## Discussion

To our knowledge, this is the first study to explore the potential selection bias resulting from the presence of validation questions, which are commonly used to exclude insufficient effort responding. This study has several important findings. Firstly, inconsistent with previous studies, [16] IERPs and non-IERPs reported significantly different levels of SES in the present study. The participants who were IERPs had higher proportions of low education level and rural residence compared with Non-IERPs. Secondly, the participants who were IERPs had higher rates of antibiotic misuse intentions. Thirdly, we found that IERPs with low SES reported significantly higher rates of antibiotic misuse intentions compared with IERPs with high SES. Therefore, instead of treating IERPs as a methodological nuisance, [17, 18] as most researchers generally do, we should pay extra attention to IERPs in this circumstance.

IERPs may be due to negative attitudes toward surveys in general, [19] the inclusion of sensitive items, [20] and lengthy surveys [21]. When IERPs are truly insufficient in their effort, outcomes should be distributed uniformly,[22] which is not the case in the present study. The mixed effects between insufficient effort responding and SES in Table 4, Table 5, and Table 6 suggest that participants engaged in insufficient effort responding because of confusion or reading comprehension difficulties caused by relatively disadvantaged SES. Literacy can result from many factors, with education level being crucial [23].

Education levels of the general population vary significantly across different parts of China. People living in more developed areas of China have better reading comprehension because of the presence of higher quality education, while people living in developing areas may have poorer reading abilities. For people living in developing areas who might have reading problems, the combination of a self-administered questionnaire and the validation questions employed in the present study may not always be appropriate. Our data showed that researchers had to exercise extra caution when administering the surveys in developing areas. As a possible solution for this situation, face-to-face interviews may be more suitable than self-administered questionnaires for participants who are poorly educated or who easily get confused or have reading/comprehension difficulties when completing a questionnaire. Face-to-face interviews have traditionally been considered as the gold standard because of their ability to obtain high unit and item response rates and valid data.[24]

There are limitations to this study. Firstly, our present study is based on the assumption that IERPs offered authentic socio-economic and health-outcomes information, which may need further validation. Participants might offer authentic socio-economic information, as there were no sensitive items and anonymity was guaranteed. However, participants might hold their positions on intention towards using antibiotics to conform with social norm. Secondly, participants with limited reading ability might keep an attentive and cooperative attitude for the first few minutes, but lose patience when confronted with dozens of antibiotic knowledge items. As noted, the average insufficient effort responses are pulled toward the midpoint when the average sufficient effort responses are away from the midpoint.[23] Thus, IERPs were more likely to choose the midpoint- “No”- on their intentions to use antibiotics for self-limiting disease, which means in this study, IERPs’ rates of antibiotic misuse intentions might be underestimated.

## Conclusions

This is the first study to explore the potential selection bias caused by validation questions, which are commonly used to exclude IERPs. The present study shows that using validation question to exclude IERPs in developing areas can result in selection bias in which participants with low SES and poor antibiotic use intentions were disproportionately excluded.

## Data Availability

The data-sets analyzed during this study are available from the corresponding author on reasonable request.

## Abbreviations

IERPs: Insufficient effort responding participants
Non-IERPs: Non-insufficient effort responding participants
QR: Quick response
